# Methanol extract of *Dicranopteris linearis* L. leaves impedes acetaminophen-induced liver intoxication partly by enhancing the endogenous antioxidant system

**DOI:** 10.1186/s12906-017-1781-5

**Published:** 2017-05-18

**Authors:** Zainul Amiruddin Zakaria, Farah Hidayah Kamisan, Maizatul Hasyima Omar, Nur Diyana Mahmood, Fezah Othman, Siti Selina Abdul Hamid, Muhammad Nazrul Hakim Abdullah

**Affiliations:** 1 Halal Product Research Institute, Universiti Putra Malaysia, 43400 UPM Serdang, Selangor Malaysia; 2 Department of Biomedical Sciences, Faculty of Medicine and Health Sciences, Universiti Putra Malaysia, 43400 UPM Serdang, Selangor Malaysia; 3 Phytochemistry Unit, Herbal Medicine Research Centre, Institute for Medical Research, Jalan Pahang, 50588 Kuala Lumpur, Malaysia; 4 Medical Technology Division, Malaysian Nuclear Agency, Bangi, 43000 Kajang, Selangor Malaysia

**Keywords:** *Dicranopteris linearis*, Gleicheniaceae, Methanol extract, Hepatoprotection, Volatile compounds

## Abstract

**Background:**

The present study investigated the potential of methanolic extract of *Dicranopteris linearis* (MEDL) leaves to attenuate liver intoxication induced by acetaminophen (APAP) in rats.

**Methods:**

A group of mice (*n* = 5) treated orally with a single dose (5000 mg/kg) of MEDL was first subjected to the acute toxicity study using the OECD 420 model. In the hepatoprotective study, six groups of rats (*n* = 6) were used and each received as follows: Group 1 (normal control; pretreated with 10% DMSO (extract’s vehicle) followed by treatment with 10% DMSO (hepatotoxin’s vehicle) (10% DMSO +10% DMSO)), Group 2 (hepatotoxic control; 10% DMSO +3 g/kg APAP (hepatotoxin)), Group 3 (positive control; 200 mg/kg silymarin +3 g/kg APAP), Group 4 (50 mg/kg MEDL +3 g/kg APAP), Group 5 (250 mg/kg MEDL +3 g/kg APAP) or Group 6 (500 mg/kg MEDL +3 g/kg APAP). The test solutions pre-treatment were made orally once daily for 7 consecutive days, and 1 h after the last test solutions administration (on Day 7th), the rats were treated with vehicle or APAP. Blood were collected from those treated rats for biochemical analyses, which were then euthanized to collect their liver for endogenous antioxidant enzymes determination and histopathological examination. The extract was also subjected to in vitro anti-inflammatory investigation and, HPLC and GCMS analyses.

**Results:**

Pre-treatment of rats (Group 2) with 10% DMSO failed to attenuate the toxic effect of APAP on the liver as seen under the microscopic examination. This observation was supported by the significant (*p* < 0.05) increased in the level of serum liver enzymes of alanine transaminase (ALT), aspartate transaminase (AST) and alkaline phosphatase (ALP), and significant (*p* < 0.05) decreased in the activity of endogenous antioxidant enzymes of catalase (CAT) and superoxide dismutase (SOD) in comparison to Group 1. Pre-treatment with MEDL, at all doses, significantly (*p* < 0.05) reduced the level of ALT and AST while the levels of CAT and SOD was significantly (*p* < 0.05) restored to their normal value. Histopathological studies showed remarkable improvement in the liver cells architecture with increase in dose of the extract. MEDL also demonstrated a low to none inhibitory activity against the respective LOX- and NO-mediated inflammatory activity. The HPLC and GCMS analyses of MEDL demonstrated the presence of several non-volatile (such as rutin, gallic acid etc.) and volatile (such as methyl palmitate, shikimic acid etc.) bioactive compounds.

**Conclusion:**

MEDL exerts hepatoprotective activity against APAP-induced intoxication possibly via its ability to partly activate the endogenous antioxidant system and presence of various volatile and non-volatile bioactive compounds that might act synergistically to enhance the hepatoprotective effect.

## Background

Medicinal plants have been the subjects of man’s interest since a long time ago with almost every culture has a history of plants’ use to treat various diseases [1]. Approximately 80% of the people in the developing countries still depend on medicinal plants as their main source of health care needs. The revival of public awareness in plant-based medicine combined with rapid expansion of pharmaceutical industries in the recent couple of years has imposed an increase demand for medicinal plants [2]. This is further supported by the fact that approximately 80% of claimed traditional medicine involves the use of plant extracts, which suggests that the medicinal plants still continue to occupy an important niche in modern medicine and may play a leading role in the introduction of new therapeutic agents [3]. A number of medicinally-effective compounds currently used as modern drugs are derived from medicinal plants, which may consist of complex structures. Thus, synthesizing these bioactive compounds preferably in minimal chemical steps and at low budget is sometimes not accessible. Therefore, the use of plant-based extracts, either as whole or semi-purified for the treatment of diseases as applied in the traditional medicine practices can be seen as a reasonable and practical solution to overcome the issue. One of the medicinal fields where the application of medicinal plants as an alternative medicine has gained remarkable attention is related to the treatment of liver diseases or damages [4].

Liver is important to the body as it helps to regulate astounding range of imperative physiological functions that help in the maintenance, performance and regulating homeostasis of the body. Being involved in the metabolism processes, the liver is usually exposed to various types of endogenous and exogenous substances including those with toxic potentials. Normally the liver will detoxify and excrete these substances from the body. However, over or continuous exposure of the liver to toxic chemicals can inflict damages to the liver because of the release of free radicals (i.e. reactive oxygen species (ROS) or reactive nitrogen species (RNS)) resulting from the metabolisms of the toxic chemicals. The ROS or RNS, on the other hand, act by causing damage to the membrane of hepatic cells found mainly in the liver parenchyma cells resulting in several repercussions on human health. Liver damage is any circumstance that may cause liver inflammation or tissue injury and affects liver function. Liver disease badly affects over 10% of the world population [3].

Drugs have been one of the important causes of liver injury. In the United States, approximately 2000 cases of acute liver failure occur annually and drugs account for over 50% of them (37% are due to acetaminophen, 13% are idiosyncratic reactions due to other medications). Meanwhile, drugs also account for 2–5% of cases of patients hospitalized with jaundice and approximately 10% of all cases of acute hepatitis. Of these, acetaminophen (APAP) has been widely associated with liver intoxication. It is a commonly used drug to treat pain and fever relief [5]. APAP is commonly considered as a “safe drug” when taken within the suggested therapeutic dose because it can be purchased easily without prescriptions. However, it can be hepatotoxic when an overdose is administered. Accidental or intentional overdose with acetaminophen (APAP) mainly cause for the acute liver failure case. Overdose of APAP cause the generation of free radicals results in the depletion of glutathione and also cause dose-related hepatocellular necrosis [6].

Although a number of alternative treatments are available to cure most kind of the liver damages cases, several types remain untreatable and the emergence of drug resistance is persistent [1, 3]. Hence, novel treatment approaches are crucial to improve therapeutic outcome. Almost half of the agents currently used in liver therapy derived from either natural products or derivatives of natural products with plants being the main contributor [3]. One of the plants that are currently being investigated for its hepatoprotective potential is *Dicranopteris linearis* L. This plant belongs to the family Gleicheniaceae and, despite its limited medicinal uses among the Malays [7], it has been scientifically proven to exert antinociceptive, anti-inflammatory and antipyretic [8, 9], antiproliferative and antioxidant [10], and chemopreventive [11] activities. We have recently reported on the hepatoprotective activity of methanol extract of *D. linearis* against the CCl_4_-induced liver injury model [12].

Taking into account the ability of MEDL to exert antioxidant and anti-inflammatory activities [8–10] which are important in the augmentation of liver protection against damages [13], and the fact that CCl_4_–induced a different mechanisms of liver intoxication in comparison to APAP, the present study was proposed to establish the hepatoprotective activity of MEDL against APAP-induced liver damage model. In addition, we also determined the involvement of some endogenous enzymatic antioxidant system, namely catalase (CAT) and superoxide dismutase (SOD), in the attenuation of APAP-induced hepatotoxicity by MEDL and analysed the phytoconstituents of MEDL using the ESI-UHPLC and GCMS methods.

## Methods

### Plant material and preparation of the extract

The leaves of *D. linearis* were collected from their natural habitat around Serdang, Selangor, Malaysia, between February and March 2013, and a voucher specimen, SK 1987/11, was deposited at the Herbarium of the Institute of Bioscience, Universiti Putra Malaysia (UPM) after being authenticated by a botanist, Dr. Shamsul Khamis, attached to the institute. MEDL was prepared according to Zakaria et al. [12]. Approximately 160 g ground dried leaves were soaked three times for 24 h at room temperature with methanol in a 1:20 (*w*/*v*) ratio, and the methanol supernatant was evaporated under reduced pressure at 40 °C, resulting in a yield of 48.4 g dried and sticky methanol extract.

### Experimental animals

Adult male rats of Sprague-Dawley (180-220 g) were obtained from animal house, Faculty of Medicine and Health Sciences, UPM. The animals were kept in spacious, hygienic polyethylene cage with wood shaving bedding and maintained under standardized environmental conditions (27 ± 2 °C; 70–80% humidity; 12 h light/dark cycle) in the Animal House Unit, Faculty of Medicine and Health Sciences, UPM, for at least 48 h before use. Food and water were supplied ad libitum up to the beginning of the experiments. The rats were fasted for 48 h prior the assay, and 200 mg/kg of Silymarin was used as the standard drug and the extract were administered orally with 10% dimethyl sulfoxide (DMSO; 10 ml/kg) as the vehicle. The study protocol of the present study was approved by the Animal House and Use Committee, Faculty of Medicine and Health Sciences, UPM (Ethical approval no.: UPM/FPSK/PADS/BR-UUH/00449). The rats were handled in accordance with current UPM guidelines for the care of laboratory animals and the ethical guidelines for investigations of experimental pain in conscious animals. All experiments were conducted between 09.30 and 18.30 h to minimize the effects of environmental changes.

### Acute toxicity study

The acute toxicity study of MEDL was performed according to the Organisation for Economic Cooperation and Development (OECD) 420 guideline for acute toxicity testing [14] with slight modification using a single-dose administration of 5000 mg/kg (orally) in five healthy male and female mice each. The control group received only the vehicle (10% dimethyl sulfoxide; DMSO; Fisher Scientific, UK). Mice were fasted overnight, prior to study and weighed before the administration of MEDL. The effects of a single oral dose of MEDL were monitored over a 14-day period for any clinical and mortality signs. The body weight of the mice was weighed on the 1st, 7th, and 14th day. At day 15, all animals were anesthetized by ketamine (100 mg/kg; intramuscular (i.m.)) and xylazine (16 mg/kg; i.m.) and then, blood samples were collected by cardiac puncture for haematological and biochemical analysis. Then, mice were sacrificed by cervical dislocation, and the vital organs were excised and weighed to determine relative organ weights (ROW). The ROW of each organ was then calculated as follows:

$$ \mathrm{ROW}=\left[\mathrm{Liver}\ \mathrm{weight}\ \left(\mathrm{LW}\right)\ \mathrm{of}\ \mathrm{rat}/\mathrm{Body}\ \mathrm{weight}\ \mathrm{of}\ \mathrm{rat}\;(\mathrm{BW})\right]\times 100\% $$

The LD_50_ was predicted to be above 5000 mg/kg if three or more mice survived.

### Hepatoprotective assay

In order to assess hepatoprotective activity of MEDL in the experimental rats, the animals were divided into the following groups by randomisation and each groups containing 6 rats (*n* = 6):

- **Group 1**: Control rats: served as normal control and received only 10% DMSO by orally (p.o.).
- **Group 2**: APAP treated rats: 10% DMSO p.o. for 7 days + APAP 3 g/kg p.o. at day 7th.
- **Group 3**: Reference rats: treated with 200 mg/kg Silymarin p.o. for 7 days + APAP 3 g/kg body weight p.o. on day 7th.
- **Group 4**: Extract treated rats: received MEDL 50 mg/kg p.o. for 7 days + APAP 3 g/kg p.o. on day 7th.
- **Group 5**: Extract treated rats: received MEDL 250 mg/kg p.o. for 7 days + APAP 3 g/kg p.o. on day 7th.
- **Group 6**: Extract treated rats: received MEDL 500 mg/kg p.o. for 7 days + APAP 3 g/kg p.o. on day 7th.

After 48 h of the last treatment, the rats were anaesthetized with diethyl ether and blood samples from each animal of all groups were collected by cardiac puncture in sterilized centrifuge tubes. After that, the rats were sacrificed and the liver was immediately removed. Livers washed in ice- cold saline, a section from the median lobe was fixed in 10% formalin for microscopic analysis and the remaining liver was quickly frozen in dry ice and stored at −80 °C for further analysis [12].

### Microscopic analysis of liver of hepatotoxic rats pre-treated with crude MEDL

After the liver tissue was fixed in 10% formalin, specimens were embedded in paraffin, sectioned (3–5 μm), and stained with hematoxylin and eosin. The histochemical sections were evaluated under the light microscope with the help and guide by a certified pathologist according to the severity of hepatic injury as described by El-Beshbishy et al. [15] with modifications.

### Biochemical analysis of blood serum liver enzymes of hepatotoxic rats pre-treated with crude MEDL

Biochemical parameters were assayed according to standard methods [12]. Plasma portion was separated from each blood sample by centrifugation at 3000 rpm for 10 min and subjected to biochemical analysis to assess liver function on the basis of serum alanine transaminase (ALT), aspartate transaminase (AST), and alkaline phosphatase (ALP). Those enzymes were measured using the Hitachi 902 Automatic Chemical Analyser.

### Determination of antioxidant enzymes level in liver homogenates of hepatotoxic rats pre-treated with crude MEDL

#### Preparation of liver homogenates

Liver homogenates were prepared by mincing and homogenizing approximately 100 mg of liver tissue in 1 mL cold PBS buffer with a steel homogenizer. The homogenate was centrifuged using Thermo Scientific centrifuge (Legend Micro 17 R) at 4000 rpm and 4 °C for 25 min. The resultant supernatant was used for the determination of enzymes’ activities [12].

#### Measurement of superoxide dismutase (SOD) and glutathione (GSH) levels, and catalase (CAT) activity

Liver tissues of the rats pre-treated with vehicle (10% DMSO), Silymarin (200 mg/kg) or MEDL (50, 250 and 500 mg/kg) followed by liver injury induction using Acetaminophen (3 g/kg) were used for the determination of SOD, GSH level and CAT activity. Liver tissue was cut into pieces and the exact weight was recorded. The tissues were homogenized with a homogenizer using appropriate cold buffer and then were centrifuged at 10000 g for 15 min at 4 °C. The supernatants were used to determine the activities of CAT and levels of SOD, and GSH. The concentration of protein in the supernatants was measured by the Bradford method using bovine serum albumin (BSA) as a standard. Levels of SOD, GSH and CAT were determined using the commercial assay kits according to the manufacturer’s instructions, respectively (Superoxide Dismutase Assay Kit, Glutathione Assay Kit and Catalase Assay Kit, Cayman Chemical Company, Ann Arbor, MI, US).

#### In vitro anti-inflammatory effect of MEDL

##### 2.7.3.1.Lipoxygenase assay

The lipoxygenase (LOX) assay was measured using spectrophotometric method as described by Azhar-Ul-Haq et al. [16]. 160 ml of sodium phosphate buffer (0.1 M, pH 8.0), 10 ml of MEDL and 20 ml of soybean LOX solution were mixed and then incubated for 10 min at 25 °C. 10 ml of the substrate in the form of sodium linoleic acid of solution was added to initiate the reaction. The enzymatic conversion of linoleic acid to form (9Z,11E)-(13S)- 13-hydroperoxyoctadeca-9,11-dienoate was followed by the change of absorbance measured at 234 nm over the period of 6 min. Reference standards and MEDL were dissolved in methanol. All reactions were completed in triplicates in a 96-well microplate.

##### 2.7.3.2.Xanthine oxidase assay

The spectrophotometric method described by Orhan et al. [17] was adopted to measure the xanthine oxidase (XO) inhibiting activity of MEDL. Approximately 10 μl of the test solution and 10 μl of XO solution were mixed with 130 μl of potassium phosphate buffer (0.05 M, pH 7.5) and incubated for 10 min at 25 °C. The addition of 100 μl of the substrate in the form of xanthine solution was done to initiate the reaction. The enzymatic conversion of xanthine to form uric acid and hydrogen peroxides measure data absorbance of 295 nm. Reference standard and MEDL were dissolved in DMSO. All reactions were completed in triplicates in a 96-well UV microplate.

### GC–MS analysis of crude MEDL

GC-MS analysis of MEDL were performed using an Agilent GC system (Model no. Agilent 19091S-433) attached to the gas chromatograph-interfaced to a mass spectrometer detector (GC-MSD) equipped with a HP-5MS silica capillary column (30.0 m X 250 μm X 0.25 μm nominal), composed of 5% phenyl methyl siloxane. For GC-MS detection, an electron ionization system with ionizing energy of 70 eV was used. Helium gas (99.999%) was used as the carrier gas at constant flow rate of 1 mL/min and an injection volume of 1 μL was employed (split ratio of 10:1); mode Split-Splitless Inlet; Injector temperature 250 °C (pressure 10.39 psi); Ion-source temperature 280 °C. The oven temperature was programmed from 100 °C (isothermal for 2 min.) and maximum oven configuration at 325 °C, with an increase of 10 °C/min, to 200 °C, then 5 °C/min to 280 °C, ending with a 9 min isothermal at 280 °C. Mass spectra were taken at 70 eV; a scan interval of 0.5 s and fragments from 45 to 450 Da. Total GC running time was 35.50 min. The relative % amount of each component was calculated by comparing its average peak area to the total areas, software adopted to handle mass spectra and chromatograms was a Turbomass. For identification of compounds, the interpretation on mass spectrum GC-MS was conducted using the database of National Institute Standard and technology (NIST) having more than 62,000 patterns. The spectrum of the unknown component was compared with the spectrum of the known components stored in the NIST library. The name, molecular weight and structure of the components of the test materials were ascertained.

### UPLC-ESI/HRMS analysis of crude MEDL

The UPLC-HRMS system used consisted of an Orbitrap with an pump, thermo scientific autosampler (ThermoFisher Scientific, USA) and Dionex column compartment. Separation was carried out on C18 Cortecs column (100 mm × 2.1 mm i.d. 1.6 μm Waters) at a flow rate 0.2 mL/min. The mobile phase consisted of a combination A (0.1% Aqueous formic acid, *v*/v) and B (100% acetonitrile). The linear gradient was from 5 to 40% B (*v*/v) at 50 min. The negative ionization mode was used and the conditions were set as follows: sheath gas at 15 (arbitray units), aux at 20 and sweep gas at 5 (arbitrary units), spray voltage at 3.0 kV, capillary temperature at 350 °C, and s-lens RF level at 55 V. The mass range was from 100 to 1000 amu with a resolution of 17,000, FTMS AGC target at 2e5, FT-MS/MS AGC target at 1e5, isolation width of 1.5 amu, and max ion injection time of 500 ms and the normalization collision energy at 35%. Throughout the analysis, reverse osmosis Milli-Q water (18.2 MΩ) (Millipore, Billerica, USA) was used for all solutions and dilutions. Chemical standards such as, gallic acid, ferulic acid, quercetin, catechin, rutin, kaempferol, apigenin and apigenin-7-O-glucoside were purchased from Sigma Co. (USA) while acetonitrile and formic acid of LCMS grade were obtained from Fisher Scientific (M) Sdn. Bhd (Kuala Lumpur, Malaysia). The standards were diluted in methanol/water, (v:v, 1:1) to 10 mg/mL and filtered through 0.22 μm membranes prior to LC-MS analysis.

### Statistical analysis

The result were expressed as means ± standard error mean (SEM) and values were calculated for each group. A one way analysis of variance (ANOVA) followed by the Tukey’s post hoc test was performed for significance analysis using Graph Pad Prism software. The minimum level of significance was set of *P* < 0.05.

## Results

### Acute toxicity observations

#### Effect of acute oral administration of MEDL on the mortality and morbidity in mice

**Fig. 1:**
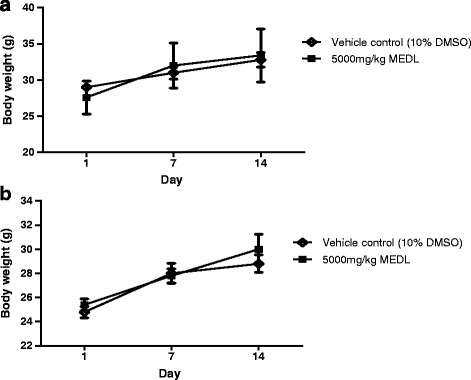
Effect of MEDL on body weight of male (**a**) and female (**b**) ICR mice in acute oral toxicity. Values are expressed as mean ± S.E.M. (*n* = 5/group)

All the male and female mice survived throughout the observing period of 14 days, and also did not exhibit any toxic clinical signs or changes in behavioural pattern. Body weights of animals in both sexes were recorded in Fig. 1. There were no significant difference between body weight and ROWs of the 5000 mg/kg MEDL-treated group when compared to the control group (vehicle-treated) for both sexes.

#### Effect of acute oral administration of MEDL on the haematological and biochemical parameters in mice

The analyzed haematological parameters, namely total red blood cell (RBC), haemoglobin concentration (Hb), packed cell volume (PCV), mean corpuscular volume (MCV), mean corpuscular haemoglobin concentration (MCHC), platelets count and total white blood cell (WBC), for male ICR mice treated with 5000 mg/kg MEDL were not significantly different when compared to the control mice (vehicle-treated) (Table 1). In contrast, MCV and total WBC of female ICR mice treated with 5000 mg/kg MEDL significantly different (*p* < 0.05) when compared to the control mice. However, the value for those parameters is still within the normal range value. The biochemical analyses indicated that there were no significant differences detected for any of the parameters for either the control or MEDL-treated group except for total bilirubin and creatinine level of male and female mice and AST level of female mice (Table 2).

**Table 1:** Haematology value of rats treated with single dose of *Dicranopteris linearis* extract

Hematological parameters		Treatment	
		Control	5000 mg/kg MEDL
Male			
Haemoglobin	g/L	133.80 ± 2.99	144.60 ± 7.53
Total red blood cell	×10^12^/L	8.49 ± 0.08	8.75 ± 0.49
Total white blood cell	×10^9^/L	5.62 ± 0.68	8.06 ± 0.96
Packed cell volume	L/L	0.37 ± 0.01	0.36 ± 0.02
Mean corpuscular volume	fL	43.80 ± 0.74	41.40 ± 1.25
Mean corpuscular Hb conc	g/L	352.20 ± 3.40	369.00 ± 14.73
Platelet count	×10^9^/L	1365.00 ± 90.28	1691.00 ± 240.20
Female			
Haemoglobin	g/L	137.60 ± 3.44	141.40 ± 5.80
Total red blood cell	×10^12^/L	8.00 ± 0.26	8.05 ± 0.20
Total white blood cell	×10^9^/L	2.64 ± 0.46	5.50 ± 0.68*
Packed cell volume	L/L	0.35 ± 0.01	0.32 ± 0.01
Mean corpuscular volume	fL	43.60 ± 1.03	40.20 ± 0.80*
Mean corpuscular Hb conc	g/L	394.00 ± 13.11	406.80 ± 7.32
Platelet count	×10^9^/L	537.60 ± 131.50	363.20 ± 210.30

Values are mean ± S.E.M. for 5 rats in each group

^*^Statistically significant compared to control (*P* < 0.05)

**Table 2:** Biochemistry value of rats treated with single dose of *Dicranopteris linearis* methanol extract

Biochemical parameters		Treatment	
		Control	5000 mg/kg MEDL
Male			
Total protein	g/L	50.62 ± 1.49	58.42 ± 2.06
Total bilirubin	umol/L	0.14 ± 0.02	1.30 ± 0.44
Alkaline phosphatase (ALP)	U/L	141.40 ± 14.82	156.80 ± 9.69*
Alanine transaminase (ALT)	U/L	169.40 ± 39.95	201.70 ± 31.42
Aspartate transaminase (AST)	U/L	623.90 ± 246.20	801.40 ± 126.90
Creatinine	umol/L	28.20 ± 1.63	46.40 ± 3.01*
Female			
Total protein	g/L	54.96 ± 2.38	48.86 ± 2.26
Total bilirubin	umol/L	0.14 ± 0.02	1.38 ± 0.42*
Alkaline phosphatase (ALP)	U/L	133.20 ± 17.71	177.00 ± 25.20
Alanine transaminase (ALT)	U/L	95.06 ± 17.71	101.60 ± 25.78
Aspartate transaminase (AST)	U/L	169.70 ± 30.53	552.40 ± 54.31*
Creatinine	umol/L	25.60 ± 0.81	47.80 ± 2.44*

Values are mean ± S.E.M. for 5 rats in each group

^*^Statistically significant compared to control (*P* < 0.05)

#### Histopathological study on various organs following the acute oral administration of MEDL in mice

**Fig. 2:**
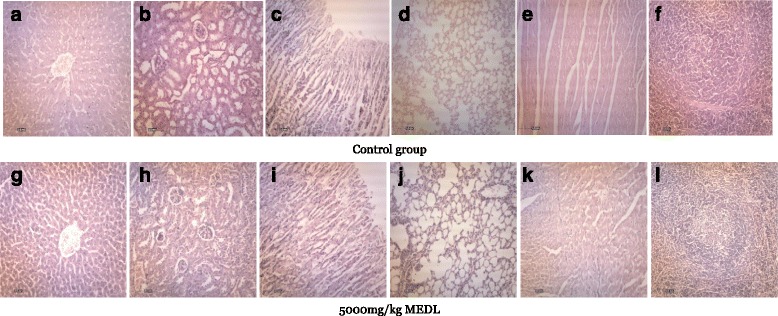
Photomicrographs of different organ from representative male ICR mice in acute oral toxicity: **a**) and **g**): liver, **b**) and **h**): kidney, **c**) and **i**): stomach, **d**) and **j**): lung, **e**) and **k**): heart, and **f**) and **l**): spleen, stained with haematoxylin and eosin (100×)

Figure 2 presents the overview histology of the liver, kidney, spleen, lung, stomach and heart of all tested group. There were no lesions or pathological changes in those organs for either sex that can be attributable to the administration of MEDL.

### Hepatoprotective effect of MEDL

#### Effects of MEDL on the body and liver weights of APAP intoxicated rats

Table 3 shows the body and liver weights of rats treated with APAP following pretreatment with MEDL or silymarin. The rats intoxicated with APAP exhibited significant (*P* < 0.05) increase in liver weight and liver/body weight ratio when compared to rats in the normal control group. Pre-administration of MEDL, at all doses, significantly (*P* < 0.05) reduced the liver weight and liver/body weight ratio of the APAP-treated rats. In addition, pre-administration of silymarin also caused significant (*P* < 0.05) reduction in the liver weight and liver/body weight ratio of the hepatotoxic rats, which was comparable to the effects observed in group pre-treated with 50 mg/kg MEDL.

**Table 3:** Effect of MEDL on body weight and liver weight in experimental rats

Treatment	Dose (mg/kg)	Body weight (BW) (g)	Liver weight (LW) (g)	LW/BW (%)
Control	-	208.70 ± 5.55	5. 85 ± 0.29	2.80 ± 0.07
APAP treated rats		219.50 ± 4.72	9.72 ± 0.15^a^	4. 44 ± 0.43^a^
Silymarin + APAP	200	200.00 ± 4.67	6.94 ± 0.24^ab^	3.47 ± 0.11^ab^
MEDL + APAP	50	218.07 ± 3.19	7.83 ± 0.88^ab^	3.59 ± 0.80^ab^
	250	224.10 ± 3.41	6.16 ± 0.51^b^	2.75 ± 0.87^b^
	500	218.64 ± 2.83	6.65 ± 0.87^b^	3.04 ± 0.88^b^

Values are expressed as means ± S.E.M. of six replicates

^a^Significant different as compared to normal control, *p* < 0.05

^b^Significant different as compared to negative control, *p* < 0.05

#### Effects of MEDL on the level of serum liver enzymes of APAP intoxicated rats

Administration of APAP had resulted in hepatotoxicity, as evident by the significant (*p* < 0.05) increase in biochemical parameters. The methanolic extract of *D. linearis* leaves, at all doses, caused significant (*p* < 0.05) reduction in the elevated levels of serum liver enzymes selected for the study. The results are summarized as Table 4.

**Table 4:** Effects of different treatments on serum liver biomarkers of experimental rats

Treatment	Dose (mg/kg)	ALT (U/L)	AST (U/L)	ALP (U/L)
Control	-	158.30 ± 2.86	95.13 ± 5.92	115.70 ± 6.99
APAP control (neg)		1714.00 ± 142.20^a^	2266.00 ± 340.40^a^	330.00 ± 42.35^a^
Silymarin +APAP (pos)	200	474.50 ± 82.17^b^	690.90 ± 146.60^b^	195.50 ± 11.06^b^
MEDL	50	225.90 ± 77.18^b^	309.50 ± 94.85^b^	252.20 ± 45.72
	250	479.00 ± 204.10^b^	1184.00 ± 564.30^b^	228.20 ± 36.76^b^
	500	445.10 ± 66.73^b^	786.60 ± 96.77^b^	350.30 ± 41.33

Values are expressed as means ± S.E.M. of six replicates

^a^Significant different as compared to normal control, *p* < 0.05

^b^Significant different as compared to negative control, *p* < 0.05

#### Effect of MEDL on the role of SOD and GSH levels, and CAT activity in liver homogenates intoxicated with APAP

The level of SOD and GSH, and the activity of CAT in liver tissue of APAP-intoxicated rats administered with MEDL are shown Table 5. From the results obtained, it was found that the hepatotoxic group (negative) caused significant (*p* < 0.05) decreased in the level of activity of SOD and CAT when compared to the normal group. Silymarin and MEDL, at various doses, significantly (*p* < 0.05) reversed the effect of APAP on liver’s antioxidant enzymes by increasing the activities towards normal value.

**Table 5:** Antioxidant enzyme activities in liver tissue of APAP- treated rats, SOD (U/g tissue) and CAT (U/g tissue)

Treatment	Dose (mg/kg)	SOD (U/g tissue)	CAT (U/g tissue)	GSH (μM/mg protein)
Normal	-	9.66 ± 0.38	114.80 ± 1.63	68.32 ± 1.19
10% DMSO + APAP	-	3.99 ± 0.07^a^	91.36 ± 1.27^a^	29.77 ± 0.47
Silymarin + APAP	200	15.37 ± 0.35^b^	109.50 ± 4.69^b^	59.44 ± 0.95
MEDL + APAP	50	13.04 ± 0.16^b^	115.40 ± 2.37^b^	38.12 ± 0.71
	250	11.29 ± 0.13^b^	120.30 ± 1.09^b^	53.11 ± 1.05
	500	11.57 ± 0.33^b^	118.2 ± 0.81^b^	63.83 ± 2.03

Values are expressed as means ± S.E.M. of six replicates

^a^Significant different as compared to normal control, *P* < 0.05

^b^Significant different as compared to negative control (10% DMSO + APAP), *P* < 0.05

#### Microsocpic findings on effect of MEDL on APAP intoxicated liver

**Fig. 3:**
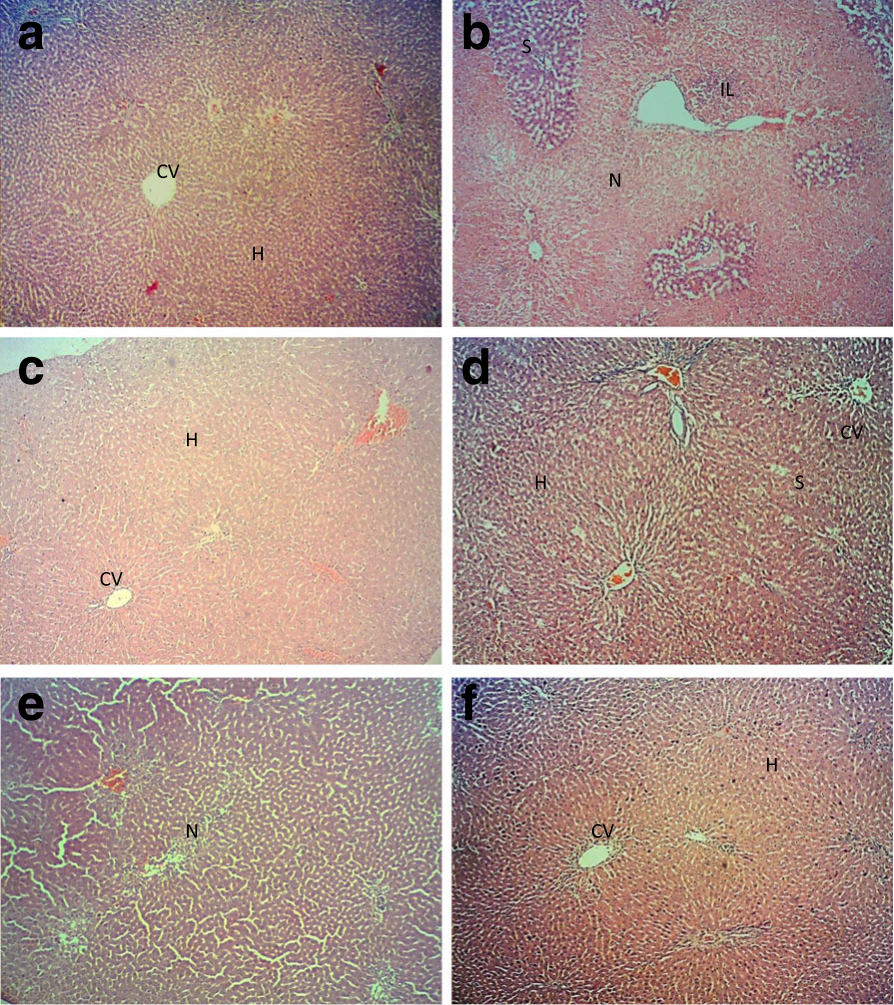
Liver photomicrographs. **a**) Normal architecture of liver showed the central vein (CV) and hepatocytes (H). **b**) APAP- treated group, showed necrosis of the hepatocytes (N), steatosis (S) and also infiltration the inflammatory cells (IL). **c**) APAP- induced after pre-treatment with 200 mg/kg of Silymarin showed normal architecture of hepatocytes with mild microsteatosis. **d**) Pre-treatment with 50 mg/kg of MEDL also attenuated the histopathological changes by the APAP- induced hepatotoxicity showed mild steatosis. **e**) Pre-treatment with 250 mg/kg of MEDL showed moderate necrosis of the hepatocytes. **f**) APAP-induced hepatotoxic liver after pre-treatment with 500 mg/kg MEDL showing mild steatosis

From the histopathological analysis, the normal liver architecture where the cells were arranged radially as seen in the normal control group rats (Fig. 3a), were disturbed following the administration of APAP. In the negative control group rats, the damage liver architecture demonstrated the presence of fatty degeneration and vacuole formation with some of the hepatocytes found to be in the necrosis state (Fig. 3b). In rats pre-treated with 200 mg/kg of silymarin (p.o.), the liver appeared to show normal architecture indicating hepatoprotection by silymarin against the damage effect of APAP (Fig. 3c). The liver dissected from the rats pre-treated with 50 mg/kg body weight MEDL (p.o.) followed by the APAP treatment showed the presence of vacuole formation, but the damage to the cells has been attenuated (Fig. 3d). Similar architecture and cell arrangement as seen with silymarin pre-treated group were observed in the liver sections of rats pre-treated with the high doses (250 and 500 mg/kg; p.o.) of MEDL (Fig. 3e and f). These alterations in the liver architecture were coincided with the corresponding changes in the serum liver enzymes level and hence the hepatoprotective effect of *Dicranopteris linearis* extract were confirmed.

#### In vitro anti-inflammatory activity of MEDL

At 100 μg/ml, MEDL induced a low inhibitory effect (18.98 ± 2.68%) against the LOX activity with no activity recorded against XO.

### Phytochemical analyses of MEDL

#### UHPLC-ESI/HRMS profile of crude MEDL

**Fig. 4:**
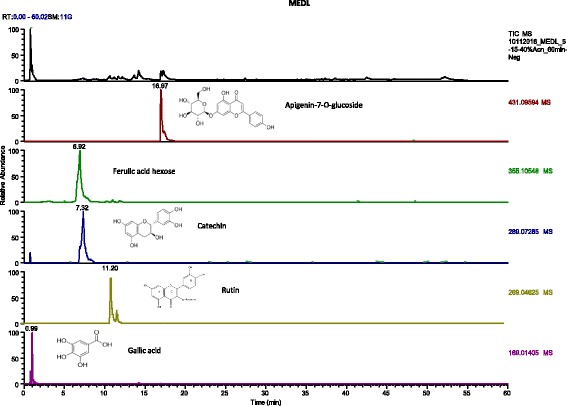
UHPLC-ESI-HRMS chromatogram of MEDL in comparison to several traces of flavonoids identified in it

*D. linearis* extract was analysed based on the accurate mass data of the molecular ions, in which ions detected were tentatively identified by their generated molecular formula, through the software Data analysis (Xcalibur) which provided list of possible elemental formulas, together with the use of standard when available and after thorough survey of the literature*.* Following the analysis, apigenin-7-O-glucoside, ferulic acid hexose, catechin, rutin and gallic acid were detected in MEDL (Fig. 4).

#### GC-MS profile of crude MEDL

**Fig. 5:**
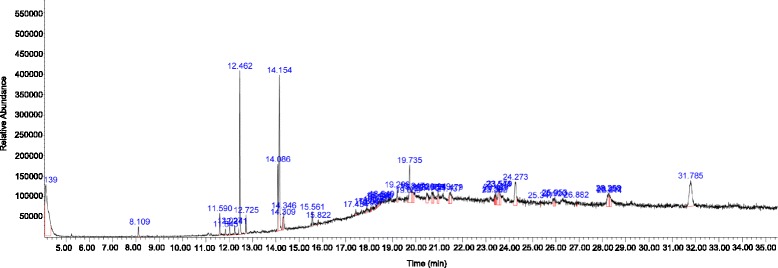
GCMS chromatogram shows the presence of at least 48 volatile compounds in MEDL

GC-MS spectra profile of crude MEDL is presented in Fig. 5 while the identified volatile compounds are presented in Table 6. Fourty eight volatile compounds were identified in MEDL with triphenylphosphine oxide (17.52%), methyl-9,12,15-octadecatrienoate (13.43%), methyl palmitate (9.70%), 3,4-Pyridinedicarboxylic acid, 6-(4-chlorophenyl)-, dimethyl ester (7.98%), erucylamide (5.45%), 5,10-Dihexyl-5,10-diihydroindolo[3,2-b]indole-2,7-dicarbaldehyde (4.63%) and methyl linoleate (4.17%) identified as the major volatile compounds. Several of these compounds have been reported to exert anti-inflammatory and antioxidant activities (Table 7).

**Table 6:** GCMS profile shows the volatile phytoconstiutents of MEDL

No. of peak	Retention time (min)	Area (%)	Name of the compound
1	4.139	17.52	Triphenylphosphine oxide
2	8.111	0.79	Phenol, 2,6-bis(1,1-dimethylethyl)
3	11.592	1.52	2-Hexadecen-1-ol, 3,7,11,15-tetramethyl-, [R-[R*,R*-(E)]]-
4	11.843	0.43	1,3-Propanediol, 2,2-dibromo-
5	12.026	1.68	Benzo[a]naphthacene
6	12.243	0.82	Methyl hexadecatrienoate
7	12.460	9.70	Methyl palmitate
8	12.723	1.10	Methyl 3-(3,5-di-tert-butyl-4-hydroxyphen yl)propionate
9	14.083	4.17	Methyl linoleate
10	14.152	13.43	Methyl 9,12,15- octadecatrienoate
11	14.306	0.96	trans-Phytol
12	14.346	1.69	Methyl stearate
13	15.558	0.94	(5E,7E)-Dodecadienal
14	15.821	0.26	1,1,3,3,5,5,7,7,9,9,11,11,13,13-te tradecamethyl-heptasiloxane
15	17.433	0.36	2-Myristynoyl-glycinamide
16	17.896	0.55	1,2-Benzisothiazole-3-acetic acid, methyl ester N-Methyl-1-adamantaneacetamide
17	18.067	0.25	Cyclotrisiloxane, hexamethyl- Indole-2-one, 2,3-dihydro-N-hydroxy-4-methoxy-3,3-dimethyl-
18	18.084	0.27	2,3,4-Trimethoxyphenylacetonitrile Demecolcine
19	18.290	0.22	1,1,3,3,5,5,7,7,9,9,11,11,13,13-te tradecamethyl-heptasiloxane
20	18.399	0.45	1,1,3,3,5,5,7,7,9,9, 11,11-dodecamethyl- hexasiloxane
21	18.427	0.45	4,5-Dimethyl-2,2-diphenyl-2H-imidazole
22	18.490	0.22	Silicic acid, diethyl bis(trimethylsilyl) ester Anthracene, 9-ethyl-9,10-dihydro-10-t-butyl-
23	18.541	0.25	Silane, trimethyl[5-methyl-2-(1-methylethyl)phenoxy]-
24	19.210	1.08	1,3,5-Triazine, 2-chloro-4,6-bis(methylthio)-
25	19.667	0.39	Gibberellin A3
26	19.736	5,45	Erucylamide
27	19.844	1.37	N-Methyl-1-adamantaneacetamide
28	19.896	1.02	2,4,6-Cycloheptatrien-1-one, 3,5-bis-trimethylsilyl-
29	20.467	1.79	3,3-Diisopropoxy-1,1,1,5,5,5-hexamethyltrisiloxane
30	20.685	1.04	Shikimic acid
31	20.753	1.16	1,2-Benzenediol, 3,5-bis(1,1-dimethylethyl)-
32	20.948	1.73	Merochlorophaeic Acid
33	21.439	0.87	1,3-dimethyl-4-azaphenanthrene
34	21.479	1.99	1-Amino-1-ortho-chlorophenyl-2-(2-quinoxalinyl)ethane
35	23.382	0.35	2-(Acetoxymethyl)-3-(methoxycarbonyl)biphenylene
36	23.399	0.42	1,2-Dihydroanthra[1,2-d]thiazole-2,6,11-trione
37	23.428	0.70	1,2-Bis(trimethylsilyl)benzene
38	23.542	2.37	1,3-Bis(trimethylsilyl)benzene
39	23.577	2.02	2-Methyl-3-phenylindole
40	24.274	4.63	5,10-Dihexyl-5,10-diihydroindolo[3,2-b]indole-2,7-dicarbaldehyde
41	25.348	0.20	2-Ethylacridine
42	25.920	0.86	2,4-Cyclohexadien-1-one, 3,5-bis(1,1-dimethylethyl)-4-hydroxy-
43	25.954	0.55	1,3-Bis(trimethylsilyl)benzene
44	26.880	0.29	Gibberellic acid
45	28.257	2.58	4′ methyl-2 phenylindole
46	28.292	0.48	5′-Methyl-2′-(trimethylsiloxy)acetophenone
47	28.309	1.65	5-nitrobenzofuran-2-carboxylic acid
48	31.784	7.98	3,4-Pyridinedicarboxylic acid, 6-(4-chlorophenyl)-, dimethyl ester

**Table 7:** Volatile compounds with anti-inflammatory and antioxidant activities

Volatile compound	Reported pharmacological activity/activities^a^
Phenol, 2,6-bis(1,1-dimethylethyl)	• Anti-inflammatory [30]
2-Hexadecen-1-ol, 3,7,11,15-tetramethyl-, [R-[R*,R*-(E)]]-	• Anti-inflammatory [31]
Methyl palmitate	• Antifibrotic [32] • Anti-inflammatory [33] • Antioxidant [34]
Methyl 3-(3,5-di-tert-butyl-4-hydroxyphenyl) propionate	• Antioxidant [35]
Gibberellin A3	• Anti-inflammatory [36]
N-Methyl-1-adamantaneacetamide	• Anti-inflammatory [37]
Shikimic acid	• Anti-inflammatory [38] • Antioxidant [39]

^a^Pharmacological activity/activities – only reports related to the antioxidant and/or anti-inflammatory activities of the respective compounds was cited

## Discussion

Acetaminophen is metabolically activated to form a reactive metabolite by several types of cytochrome P450 such as cytochromes 2E1, 1A2, 3A4, and 2A6 via a direct two-electron oxidation. This reactive metabolite, *N*-acetyl-*p*-benzoquinone imine (NAPQI1), covalently binds to protein to form protein adducts. At therapeutic doses, NAPQI1 is detoxified by glutathione (GSH) to form an acetaminophen-GSH conjugate. However, at a toxic dose of APAP, total hepatic GSH is remarkably depleted resulting in the metabolite binding covalently to cysteine groups on protein to form APAP-protein adducts. Events that generate hepatocellular death following the formation of APAP-protein adducts are poorly understood. However, it is suggested that covalent binding to critical cellular proteins results in subsequent loss of activity or function and eventually cell death and lysis [18]. Mitochondrial proteins as well as proteins involved in cellular ion control have been postulated to be the primary cellular targets, which upon binding with the reactive metabolites, results in the loss of energy production. In addition, the formation of protein adducts also lead to alterations of plasma membrane ATPase activity. A number of these cellular proteins that bound to intoxicated APAP have been isolated and identified such as glutamate dehydrogenase, glutathione peroxidise, carbonic anhydrase III etc. [19]. McGill et al. [19], themselves, have reported among others the involvement of mitochondrial damage and nuclear DNA fragmentation in the APAP induced liver damage.

Other than Kamiyama et al. [20] who demonstrated that lipid peroxidation play minimal role in APAP-induced liver damage via in vitro techniques, Jaeschke et al. [21] used the in vivo model to show the less critical function of lipid peroxidation in APAP-induced liver damage. The lack of lipid peroxidation involvement in APAP-induced model might be attributed to the role played by peroxynitrite, which is a highly reactive nitrating and oxidizing species. In overdose, APAP is metabolized by cytochrome P450 to form a reactive metabolites, NAPQI, which then reacts with hepatic glutathione (GSH) causing the latter depletion by approximately 90%. In addition NAPQI binds covalently to proteins as described earlier to form APAP-cysteine adducts, which then leads to the centrilobular hepatic necrosis. Further study showed that nitrated tyrosine, protein adducts formed as a result of nitration of tyrosine by peroxynitrite, occurs in hepatic centrilobular cells wherein they co-localized in cells containing the APAP-cysteine adducts. This nitrated tyrosine are formed from the action of peroxynitrite, which is a highly reactive nitrating and oxidizing species formed by the rapid reaction of nitric oxide (NO) and superoxide. It has been reported that the activated Kupffer cells are mechanistically vital in NO and superoxide formation. However, other cellular sources of NO and superoxide may also be important. Hepatocytes and stellate cells express inducible nitric oxide synthase (iNOS) while endothelial cells constitutively express eNOS. On the other hand, various sources produce superoxide, including damaged mitochondria. It is noteworthy to highlight that intoxication with hepatotoxin that caused hepatic GSH depletion (i.e. APAP, chloroform, bromobenzene, and allyl alcohol) leads to peroxynitrite formation, which then promotes toxicity.

Although the role of free radicals remains vital in the development of APAP-induced hepatotoxicity, the pathway taken by the free radicals to induce liver cells damage is different from the one used by CCl_4._ Instead of lipid peroxidation, APAP-induced liver damage uses peroxynitrite as a mediator of hepatotoxicity. Based on this fact, it is reasonable to propose that for any compounds/extracts to be considered as suitable candidates to be developed into hepatoprotective drugs, they should possess significant free radical scavenging and antioxidant effects [22]. This proposed statement is in line with the previous study that demonstrated the ameliorative effect of aqueous extract of *Dicranopteris linearis* leaves against CCl_4_- and APAP-induced liver injury, which are believed to be dependent on the extract’s antioxidant status [23]. With regard to MEDL, it has been shown to exert remarkable free radical scavenging and antioxidant activities as confirmed by several tests [12]. The antioxidant property of MEDL could be associated with the presence of several flavonoids that have been identified as part of the phytoconstituents of MEDL, such as rutin and quercitrin [10, 12]. Previously, rutin has been reported to attenuate APAP- induced hepatotoxicity in rats [24]. Albeit no data has been found on the hepatoprotective activity of quercitrin, there is a report on the ability of isoquercitrin, which is an isomer of quercetin, to prevent APAP-induced liver damage [25]. Recent UHPLC analysis of MEDL revelaed the presence of several other bioactive compounds  that exert hepatoprotective activity in addition to rutin such as catechin [26] and gallic acid [27]. Catechin and gallic acid were also reported to possess antioxidant and anti-inflammatory activities [28, 29] and, thus, are believed to synergistically act with rutin to exert the hepatoprotective effect. In addition, several volatile compounds with reported anti-inflammatory and antioxidant activities have also been identified using the GCMS equipment in present study. These compounds, namely phenol, 2,6-bis(1,1-dimethylethyl), 2-hexadecen-1-ol, 3,7,11,15-tetramethyl-, [R-[R*,R*-(E)]]-, methyl palmitate, methyl 3-(3,5-di-tert-butyl-4-hydroxyphenyl) propionate, gibberellin A3, N-methyl-1-adamantaneacetamide and shikimic acid, are thought to synergistically contribute to the attenuation of APAP-induced liver intoxication [30–39].

The involvement of endogenous antioxidant defence mechanism in the protection of liver against oxidative stress has been greatly acknowledged [40]. As described earlier, parenchymal cells are the main site where oxidative stress took place, which if not attenuated can lead to liver injury. As part of the endogenous defence mechanisms, the liver develops its own complicated antioxidant system consisting of a number of enzymatic and non-enzymatic antioxidants that serves to protect the liver from ROS/RNS-induced damage and, at the same time, provide maintenance to the liver’s redox homeostasis. Nevertheless, the presence of too much ROS/RNS can disturb the liver’s homeostasis, which can trigger oxidative stress that can lead to liver injury [41]. The involvement of some of the members of endogenous antioxidant system, namely SOD, CAT and GSH, in the modulation of hepatoprotective activity of MEDL against APAP were analysed in the present study. From the results obtained, the function of SOD, CAT and GSH in protecting the liver against the action of ROS/RNS was reduced following intoxication by APAP. The observed reduction in function of some of the endogenous antioxidant members as seen with SOD, CAT and GSH suggested the development of liver injury. This finding was further supported by the increase in level of serum liver enzymes, particularly of ALT and AST, as well as the microscopic observation that shows destruction in the liver architecture in the group intoxicated with APAP. Concurrent with the above observations, the present study also demonstrates considerable increase in the rat’s liver weight as well as liver/body weight ratio following the liver intoxication with APAP, which might also supported the condition of liver damage. In comparison, pre-treatment with MEDL caused remarkable attenuation of toxic effect triggered by APAP administration indicated by the increase in SOD and GSH level, and CAT activity in liver homogenates. In addition to the increase in the level or activity of several enzymatic and non-enzymatic components of endogenous antioxidant system, the recovery of APAP intoxicated liver can be indicated by the reduction in the serum ALT and AST level, the rat’s liver weight as well as liver/body weight ratio and progressive recovery of the liver tissue at microscopic level towards normal architecture in a dose-dependent manner. Thus, it is plausible to suggest that the ability of MEDL to exhibit hepatoprotective activity against APAP intoxication could be attributed, in part, to its capability to modulate the action of endogenous antioxidant system, in particular, the activation of SOD, CAT and GSH [40, 41].

The anti-inflammatory potential of MEDL in vitro was tested using the LOX and XO assays. LOXs are enzymes that play role in the metabolism of leukotrienes (LTs) and are susceptible to the action of antioxidants with most of their action could be associated with the inhibition of lipid hydroperoxide formation via the scavenging of lipidoxy or lipid peroxy radicals formed in the course of enzyme peroxidation [42]. On the other hand, XO function as a main biological source of oxygen-derived free radicals that contribute to the oxidative damage of living tissues such as in the medical condition known as gout, which is distinguished by hyperuricemia that initiates uric acid deposition in the joints resulting in painful inflammation [43]. Despite the fact that the chloroform and aqueous extracts of *D. linearis* have been reported to demonstrate anti-inflammatory activities when assessed using the various animal models [8, 9], MEDL showed low inhibitory effect against LOX activity (<20% inhibition) with no activity detected against XO. These discrepancies in reports could plausibly suggest that MEDL exerts the anti-inflammatory activity via mechanisms that did not involve inhibition of LOX and XO. The extracts of *D. linearis* were earlier shown to inhibit inflammation when assessed using the carrageenan-induced paw edema assay and cotton pellet-induced granuloma assay. The former assay, in particular, signifies a conventional model of edema formation and hyperalgesia, and has been comprehensively used in the development of non-steroidal anti-inflammatory drugs as well as selective COX1–2 inhibitors. COX-2 is quickly induced in the spinal cord and other regions of the CNS following carrageenan injection in the paw [44]. According to these reports, the COX-2-mediated rise in prostaglandin (PG) E2 synthesis at the central level contributes to the severity of the inflammatory and pain responses in this model. Therefore, it can be suggested that *D. linearis* extracts exhibited anti-inflammatory activity mainly via modulation of the COX-dependent pathway.

Phytochemicals analysis of MEDL using the GCMS or UHPLC-ESI/HRMS revealed the presence of several volatile (methyl palmitate [33, 34] and shikimic acid [38, 39]) and non-volatile bioactive compounds (gallic acid [28] and catechin [29]) with reported antioxidant and/or anti-inflammatory activities. In addition, several of these compounds such as methyl palmitate [45], quercetin [46], rutin [47] and gallic acid [48] have been reported to exert hepatoprotective activity and are believed to synergistically act together in MEDL to produce the observed liver protective effect against APAP intoxication.

## Conclusion

In conclusion, MEDL exerts hepatoprotective activity against APAP-induced intoxication possibly via its ability to partly activate the endogenous antioxidant system such as SOD, CAT and GSH. The hepatoprotective potential of MEDL could also be attributed to its potential antioxidant and anti-inflammatory activities and the presence of various non-volatile bioactive compounds that might act synergistically to enhance the hepatoprotective effect.

## Abbreviations

ALP: Alkaline phosphatase
ALT: Alanine transaminase
APAP: Acetaminophen
AST: Aspartate transaminase
CAT: Catalase
CCL_4_: Carbon tetrachloride
CNS: Central nervous system
COX: Cyclooxygenase
DMSO: Dimethyl sulfoxide
eNOS: Endothelial cells constitutively express
GCMS: Gas chromatography-mass spectrometry
GSH: Glutathione
Hb: Haemoglobin concentration
i.m.: Intramuscular
iNOS: Inducible nitric oxide synthase
LOX: Lipoxygenase
LTs: Leukotrienes
MCHC: Mean corpuscular haemoglobin concentration
MCV: Mean corpuscular volume
MEDL: Methanolic extract of *Dicranopteris linearis*
NAPQI: *N*-acetyl-*p*-benzoquinone imine
NO: Nitric oxide
OECD: Organisation for Economic Cooperation and Development
p.o.: Orally
PCV: Packed cell volume
RBC: Total red blood cell
RNS: Reactive nitrogen species
ROS: Reactive oxygen species
ROW: Relative organ weight
SOD: Superoxide dismutase
*W*/*V*: Weight/Volume
WBC: Total white blood cell
XO: Xanthine oxidase
